# Spiritual Rappings

**Published:** 1853-02

**Authors:** 


*Spiritual Rappings.* — The New York Medical Times states, that in the State Lunatic Asylum, of Ohio, at Columbus, are twenty persons whose insanity is clearly traceable to spiritual rappings; and in the State Asylum, at Utica, there are nine victims of the same delusion.

We happen, every now and then, to hear of the members of the Fox family pursuing their knee-and-toe-knocking vocation in different parts of the country. If there should be a reader of our Journal in any place in which they may undertake to practice their deception, and he will take the trouble to test their spiritual functions in the simple way that was adopted in Buffalo, which is described in our exposition, (vol. vi, page 628,) he will very shortly put a stop to the delusion *in that place.*

